# PRIME: A Programme to Reduce the Treatment Gap for Mental Disorders in Five Low- and Middle-Income Countries

**DOI:** 10.1371/journal.pmed.1001359

**Published:** 2012-12-27

**Authors:** Crick Lund, Mark Tomlinson, Mary De Silva, Abebaw Fekadu, Rahul Shidhaye, Mark Jordans, Inge Petersen, Arvin Bhana, Fred Kigozi, Martin Prince, Graham Thornicroft, Charlotte Hanlon, Ritsuko Kakuma, David McDaid, Shekhar Saxena, Dan Chisholm, Shoba Raja, Sarah Kippen-Wood, Simone Honikman, Lara Fairall, Vikram Patel

**Affiliations:** 1 University of Cape Town, Cape Town, South Africa; 2 Stellenbosch University, Stellenbosch, South Africa; 3 London School of Hygiene and Tropical Medicine, London, United Kingdom; 4 Addis Ababa University, Addis Ababa, Ethiopia; 5 Public Health Foundation of India, Hyderabad, India; 6 HealthNet TPO, Kathmandu, Nepal; 7 University of KwaZulu-Natal, Durban, South Africa; 8 Human Sciences Research Council, Durban, South Africa; 9 Makerere University, Kampala, Uganda; 10 Institute of Psychiatry, King's College London, London, United Kingdom; 11 University of Melbourne, Melbourne, Australia; 12 London School of Economics and Political Science, London, United Kingdom; 13 World Health Organization, Geneva, Switzerland; 14 BasicNeeds, Bangalore, India; 15 Sangath, Goa, India

## Abstract

Crick Lund and colleagues describe their plans for the PRogramme for Improving Mental health carE (PRIME), which aims to generate evidence on implementing and scaling up integrated packages of care for priority mental disorders in primary and maternal health care contexts in Ethiopia, India, Nepal, South Africa, and Uganda.

**Summary Points**

- The majority of people living with mental disorders in low- and middle-income countries do not receive the treatment that they need.
- There is an emerging evidence base for cost-effective interventions, but little is known about how these interventions can be delivered in routine primary and maternal health care settings.
- The aim of the Programme for Improving Mental Health Care (PRIME) is to generate evidence on the implementation and scaling up of integrated packages of care for priority mental disorders in primary and maternal health care contexts in Ethiopia, India, Nepal, South Africa, and Uganda.
- PRIME is working initially in one district or sub-district in each country, and integrating mental health into primary care at three levels of the health system: the health care organisation, the health facility, and the community.
- The programme is utilising the UK Medical Research Council complex interventions framework and the “theory of change” approach, incorporating a variety of qualitative and quantitative methods to evaluate the acceptability, feasibility, and impact of these packages.
- PRIME includes a strong emphasis on capacity building and the translation of research findings into policy and practice, with a view to reducing inequities and meeting the needs of vulnerable populations, particularly women and people living in poverty.

## Background

The need for mental health care services is high. More than 13% of the global burden of disease is due to neuropsychiatric disorders, and almost three-quarters of this burden lies in low- and middle-income countries (LMICs) [1]. Neuropsychiatric disorders include mental disorders (such as unipolar and bipolar affective disorders, substance use and alcohol use disorders, schizophrenia, and dementia) and neurological disorders (such as epilepsy, migraine, multiple sclerosis, and Parkinson disease) [2]. We include both these types of disorders in our broad definition of global mental health. The burden of these disorders is projected to grow dramatically in the next decade, in part because of the demographic and epidemiological transitions in LMICs [3]. However, between 76% and 84% of people with serious mental disorders (as defined by the World Health Organization [WHO] Composite International Diagnostic Instrument) in six LMICs in the World Mental Health Survey had not received treatment in the previous year [4], representing a considerable treatment gap. Where treatments are accessed, they often lack a clear evidence base and involve considerable out-of-pocket payments, which can lead to catastrophic health expenditures [5]. Budgets and human resources provided by ministries of health (MoH) for mental health care remain woefully inadequate to address the treatment gap, particularly in LMICs [6].

There is strong international consensus that narrowing the treatment gap in LMICs requires the integration of mental health into primary care, including maternal health care [7]. Such integration provides a number of advantages, including more holistic health care, increased accessibility of mental health services for people in need of care, opportunities for reducing the stigma of mental health problems by not clearly identifying patients who are receiving mental health care (which is often the case if they attend specialist facilities such as psychiatric hospitals), and reduced costs [8],[9]. There is a growing body of evidence testifying to both the efficacy of specific treatments for priority mental disorders (see Box 1) in LMICs and their cost-effectiveness [10]. This evidence has informed the policies of the WHO Mental Health Gap Action Programme (mhGAP), with its objective of scaling up services for mental, neurological, and substance use disorders [11]–[13]. Alongside mhGAP, others have developed innovative intervention models, such as maternal mental health services in the context of routine maternal care [14], livelihoods interventions for people with severe mental illness [15], and mental health interventions in complex emergencies [16],[17].

**Box 1. Guiding Principles of PRIME**

PRIME's approach is based on the following guiding principles:

1. **A focus on health systems strengthening:** The starting point for PRIME is robust evidence on which mental health care interventions need to be scaled up, but little evidence on how these interventions should be delivered in routine health care in low-resource settings. PRIME will seek to refine the knowledge on health systems interventions needed to deliver and scale up mental health care, with an emphasis on integrating care of priority mental disorders into routine primary and maternal health care.
2. **Working in partnerships:** PRIME seeks to address the knowledge gap through partnerships between academic researchers in global mental health, MoH in each study country, innovative NGOs that have developed mental health interventions in primary care and community settings, and WHO. MoH partners were involved in developing the funding proposal before the DFID grant was awarded, and care was taken to ensure that the substance of the research was aligned with MoH policy priorities.
3. **Giving priority to key mental disorders:** PRIME will focus on priority mental disorders that impose the largest burden of disease, and for which there is the most robust evidence for cost-effective and culturally acceptable interventions [10]: depression, alcohol abuse, and schizophrenia, as defined by the *International Classification of Diseases, Version 10* [30]. In addition, contextually important priority conditions have been included in site-specific plans, for example, epilepsy in Ethiopia and Uganda (epilepsy is included among the WHO mhGAP mental, neurological, and substance use disorders [11]).
4. **Use of robust frameworks for the design and evaluation of complex interventions:** The Medical Research Council framework for complex interventions [31] is the methodological basis for the development and evaluation of multi-component packages of mental health care in PRIME. The theory of change framework, drawing on theory-based programme evaluation approaches [22], will be used to develop an overarching theory of how mental health care plans can best be shaped and implemented to have an effect on the identified outcomes.
5. **Reduction of inequities:** The benefits of implementing mental health interventions should be equitably distributed, with a particular focus on outcomes in key disadvantaged groups: people living in poverty, women, and people with severe mental disorders. The goal should include reducing inequities both in access to services and in improved outcomes.

Yet evidence is still lacking on how these specific interventions can be combined into integrated packages and delivered in routine primary health care and maternal health care. Furthermore, there is limited evidence on the process and impact of scaling up such an integrated mental health care plan for a population, even at a local district level.

## Aims and Objectives of PRIME

The aim of the Programme for Improving Mental Health Care (PRIME) is to generate evidence on the implementation and scaling up of integrated packages of care for priority mental disorders in primary and maternal health care settings in Ethiopia, India, Nepal, South Africa, and Uganda. PRIME was formed in response to a call for grant proposals from the UK Department for International Development (DFID) in 2010 to establish a research programme consortium on the theme of improving mental health services in low-income countries. The PRIME consortium was awarded the grant through a competitive international tender process and began its work in May 2011.

PRIME has three objectives. (1) In the Inception phase (May 2011–March 2012), we developed draft mental health care plans, comprising packages of mental health care for delivery in primary health care and maternal health care. (2) In the Implementation phase (April 2012–March 2015), we will evaluate the feasibility, acceptability, and impact of the packages of care in primary health care and maternal health care in one low-resource district (or sub-district) in each country. (3) In the Scaling Up phase (April 2015–April 2017), we will evaluate the scaling up of these packages of care to other districts.

## Countries and Settings

PRIME will adopt the same core methodological approach in all five countries. The sites have diverse socio-cultural, urban/rural, and economic contexts, which include extremely under-resourced settings, a fragile state setting, and middle-income countries marked by high levels of socio-economic inequality (see Table 1).

**Table 1:** Country settings with district sites.

Country	District	Population	Number of Health Facilities	Socio-Economic Characteristics	Number of MH Specialists
Ethiopia	Sodo	165,000	0 hospitals, 1 district health bureau, 7 CHC, 52 HP	Literacy rate: 22%; 90% rural	None
India	Sehore (Madhya Pradesh state)	1,311,008	2 hospitals, 8 CHC, 15 PHC, 152 SHC	Literacy rate: 71%; 81% rural	1 part-time psychiatrist, 1 psychologist
Nepal	Chitwan	575,058	2 hospitals, 4 PHC, 5 HP, 41 sub-HP	Literacy rate: 70%; 73% rural	2 psychiatrists
South Africa	Kenneth Kaunda (North West Province)	632,790	4 hospitals, 1 mental hospital, 9 CHC, 28 PHC,14 mobile clinics	Literacy rate: 88%; 14% rural	1 psychiatrist, 1 psychologist
Uganda	Kamuli	740,700	2 hospitals, 41 PHC	Literacy rate: 62%; 97% rural	1 psychiatric clinical officer

CHC, community health centres; HP, health posts; PHC, primary health clinics; SHC, sub-health centres.

The specific countries chosen were selected because (1) their diverse contexts offer opportunities for adaptation of the interventions and evaluation of impacts in diverse disadvantaged populations in LMICs; (2) the lead research institutions in each country have strong, established track records demonstrating their capacity for carrying out research; and (3) these institutions have forged strong local partnerships involving MoH, other academic institutions, and non-government organisations (NGOs). The PRIME programme is founded on a number of principles (Box 1).

## Formulation of the Mental Health Care Plans

In the Inception phase, PRIME developed a draft mental health plan comprising packages of care for a unit of population, namely, the district in each country. While the goal or function of each package of the intervention is similar across settings (e.g., to improve mental health outcomes), the content or form of the package (e.g., which human resource cadre delivers the component) is informed by local needs. In this way, we will be able to describe both the impact of the intervention in each country and compare the methods used to achieve these results across countries. We will publish the country mental health care plans and the process of their development in peer-reviewed open-access journals during 2013, and the published plans will also be made available via links from our website (http://www.prime.uct.ac.za/).

**Figure 1:**
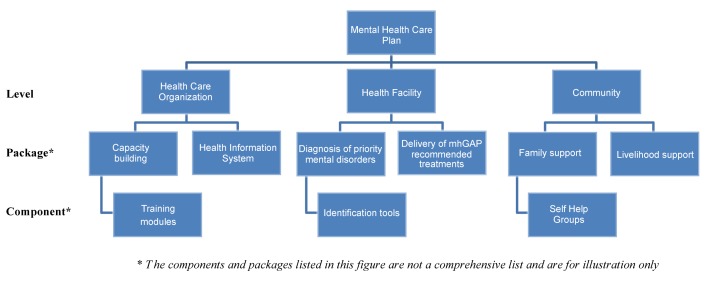
The building blocks of a mental health plan.

PRIME proposes that integrating mental health into primary care requires actions at three levels of the health system: the health care organisation, the health facility, and the community (Figure 1).

### Health Care Organisation

At the level of the health care organisation, packages include components that are relevant to organising mental health care in the district population. These include establishing the requisite governance, financing, human resources, capacity building, and information systems. The WHO mhGAP guidelines on health systems interventions and existing guidelines on mental health policy development and implementation [18],[19] will provide the basis for these packages.

### Health Care Facility

Packages at the level of the health care facility are primarily focused on the detection and treatment of mental disorders using evidence-based guidelines. The WHO mhGAP intervention guide [11], which describes the use of evidence-based treatments by non-specialist health workers in routine care settings, will form the components for the packages. People with priority disorders will be identified through a combination of community case detection using locally developed and validated tools, and primary care facility-based assessment using adaptations of the mhGAP intervention guide clinical algorithms. The delivery of these packages will be based on the evidence synthesised in the 2009 *PLoS Medicine* series on packages of care for mental disorders [20] and the WHO–World Organization of Family Doctors report on mental health in primary care [8]. This evidence base indicates that collaborative stepped care delivered by non-specialist health workers who are supervised by mental health specialists, with active participation of service users and their families, is an affordable and effective delivery system for packages of care for mental disorders [21].

### Community-Based Care

Packages at the level of the community are primarily focused on early identification, awareness raising, stigma reduction, increasing demand for appropriate mental health care, and addressing the continuing care and social and economic needs of people with priority mental disorders. People outside the formal health care system, for example, traditional healers, service users, caregivers, and community members themselves, play important complementary roles in delivering community-based care. Our NGO partners' experiences with such interventions form the basis of the community packages, and we will partner with local community-based organisations, including advocacy groups, in this aspect.

## Research Methods

We will use a range of research designs to answer our key questions, as shown in Table 2. In the Inception phase we conducted a situational analysis of the mental health system in the selected district in each country. Using these data, we engaged in formative research to refine the substance and delivery of the proposed mental health care plan. This formative work has included three aspects. (1) We conducted a series of “theory of change” consultative workshops [22]. Theory of change is a structured participatory approach to the design and evaluation of interventions that provides “a systematic and cumulative study of the links between activities, outcomes, and contexts of the initiative” ([22], p. 16). In the theory of change workshops, local stakeholders were asked to work with the research team to map out the steps in the causal pathway that lead to the intended outcome of the mental health care plan. This provided an opportunity for the research team and local stakeholders to interrogate the assumptions in each step of the proposed system change, as well as identify key indicators needed to monitor that change. (2) We conducted individual semi-structured interviews and focus group discussions to gather information from local stakeholders on the acceptability and feasibility of the proposed intervention packages. A wide range of stakeholders were interviewed, including national policy makers, district health managers, mental health specialists, primary care practitioners, community health workers, people living with the priority mental disorders, and local NGOs. Interview schedules addressed a range of topics, including experience and understanding of mental health problems, and participants' views on the draft mental health plans, training needs of primary care practitioners, task shifting, barriers to care, and health system requirements for integrating mental health into primary health care. (3) We developed a costing tool to estimate the resources required to implement the mental health care plan in each district, informed by local data and consultations.

**Table 2:** Indicative research questions, methods, and outputs for each phase of PRIME.

Questions	Methods	Outputs
**Inception phase (year 1)**		
What are the feasible and acceptable components of mental health care? How can these components be integrated into packages of care? What are the methods for integration of these packages into routine primary health care and maternal health care?	Synthesis of evidence and systematic reviews; theory of change workshops in each country; formative studies, e.g., semi-structured interviews and focus group discussions, to assess acceptability and feasibility of the packages of care	Review of interventions that break the cycle of poverty and mental illness in LMICs [29]; draft integrated mental health care plan for routine primary health care and maternal health care in each country; evidence on the acceptability and feasibility of implementing the mental health care plan
**Implementation phase (years 2–4)**		
What are the costs and impact of delivering the packages of care in routine primary health care and maternal health care settings? What are the health system requirements for scaling up—human resources, training and supervision needs, infrastructure, drugs, budgets—and the incremental cost of increasing coverage, per new patient treated? What is the impact of the integrated mental health care plan on coverage and utilisation of mental health care? How equitable is the distribution of these outcomes? What are the specific barriers that influence access to services for people living in poverty, people with severe mental disorders, and women, particularly during the perinatal period?	Costing of the components of the care package; repeated facility surveys to assess changes in detection; before–after evaluations of mental health, social, and economic outcomes in cohorts; repeated community surveys to assess changes in coverage and service utilisation	Evidence on the resources required for implementing the mental health care plan and its impact on health, social, and economic outcomes; knowledge about barriers to equitable access of services for disadvantaged populations and strategies to address these barriers; final intervention guide for each level of health care, for use in primary and maternal health care; evidence about the impact of scaling up on coverage and utilisation of mental health care, as well as the equity of coverage and utilisation
**Scaling up phase (years 4–6)**		
What is the optimal level of integration of mental health interventions in the existing primary and maternal health care system to ensure effectiveness, sustainability, quality, and coverage of services? What are the drivers and constraints to scaling up, and how can these be addressed?	Mixed methods case studies at the level of individual districts using document reviews, qualitative methods, and health management information systems data to assess health management and planning for mental health	Evidence on the optimal level of integration of mental health into primary and maternal health care in a variety of settings, as well as the residual barriers to scaling up and the strategies to address these

Once the final mental health care plan has been approved by all stakeholders, training materials will be developed, the proposed interventions will be piloted, and the intervention will then be implemented and evaluated in each district. The primary quantitative methodologies for this evaluation are influenced by recent innovations for evaluating complex interventions implemented at the level of health systems or populations. These include community-based surveys to assess changes in coverage and stigma, facility-based surveys to assess changes in case detection, case studies of district level mental health systems, and studies of cohorts of individuals treated by the mental health care plans, to assess changes in mental health, social, and economic outcomes [23]–[26]. All data will be disaggregated by gender, residence (rural/urban), and economic status to monitor equity of access to services and outcomes.

## Capacity Building

In addition to the specific research aims, a secondary aim of PRIME is to strengthen the capacity of each partner institution to generate, communicate, and utilise mental health research. We will build on the existing evidence [27] and lessons learned from other international research collaborations to strengthen individual and institutional capacity for undertaking research, disseminating research findings, and using research to guide health systems development. We envisage PRIME to be a platform of research that not only delivers specific research outputs, but also seeks additional funding to maximise opportunities and to ensure the continuation and expansion of the work beyond the tenure of the consortium. This approach seeks to strengthen individual and institutional capacity by fostering training in relevant research skills, and knowledge translation and exchange. We adopted DFID's “Ten Steps to Good Capacity Building” [28] to develop and implement our capacity building plan.

## Research Uptake

It is crucial that the research findings of PRIME are translated into policy and practice. To this end, we formulated the following specific objectives that will contribute to the uptake of our research, and to narrow the treatment gap in LMICs: (1) to increase awareness in diverse stakeholder communities, from user groups to policy makers, about the adverse impacts of mental disorders and how these can be addressed through improving access to evidence-based mental health care; (2) to mobilise people affected by mental disorders, their families, and key community stakeholders to advocate for scaling up evidence-based care for mental disorders (this will include facilitating interactions between key community stakeholders and policy makers); (3) to develop the capacity of policy makers and donors to utilise research and develop evidence-based mental health systems, integrating mental health in routine primary health care; and (4) to increase public engagement with the research findings, in particular those most affected, their families and communities, key stakeholders, and policy champions.

## Challenges in Implementation

There are a number of challenges that are likely to be faced in implementing PRIME, several of which are beyond the control of the research team. Chief among these are that MoH have limited resources to implement and scale up the mental health care plans. To address this we engaged proactively and at an early stage with our MoH partners to build realistic programmes to which MoH are willing to commit resources. This has included supporting MoH in mobilising new resources where possible, an approach that has already yielded new funding in the case of Uganda. Establishing collaborative relationships early in the process has been essential for researchers to gain an understanding of MoH policy priorities in each country, and for MoH partners to develop their ownership of the programme.

A second challenge is the risk of high staff turnover, which may undermine training and supervision interventions. To address this we aim to build capacity among local clinical staff that is appropriate to their needs, and in a manner that enables them to use their new skills in their local setting, for example, by establishing a mental health co-ordinator who oversees the available human resources in the district and ensures that an ongoing programme of training and supervision is in place.

A third challenge is that it is very difficult in real world settings to evaluate a large scale programme without having some impact on what is being evaluated, for example, through the extra resources and expertise made available by the study. We have tried to minimise this by ensuring that the interventions themselves are delivered by MoH or community-based partners in the countries, rather than by members of the PRIME research team. This is crucial for the sustainability of the programme locally, and generalisability to other settings. We will also be transparent about what additional resources and skills have been introduced through the programme, and the associated impacts, while acknowledging what may and may not be replicable in other settings.

## Conclusion: Expected Outcomes

Within the time frame of the programme, we hope to reduce the treatment gap and bring about improved mental health, social, and economic outcomes for people living with priority disorders in each district site. To assess reductions in the treatment gap in each district, we will measure changes in coverage of the priority disorders associated with implementation of the mental health care plans. Improvements in mental health, social, and economic outcomes will be assessed through repeated measures in cohorts of service users in each country site. In addition, we hope to build sustainable research capacity in participating country institutions to develop, undertake, and disseminate research on implementing and scaling up mental health services. A key outcome will be sustainable partnerships for future collaborations between the international partners and, in each country, between academic partners, MoH, and NGOs, including in other areas of the health care sector. In the longer term, PRIME hopes to achieve increased uptake of its research findings for mental health policy and practice in other regions of the study countries and other LMICs, and increased uptake by international development agencies and donors, to support scaling up of mental health care in LMICs and reduce the treatment gap for mental disorders globally.

## Abbreviations

DFID: UK Department for International Development
LMICs: low- and middle-income countries
mhGAP: Mental Health Gap Action Programme
MoH: ministries of health
NGO: non-government organisation
PRIME: Programme for Improving Mental Health Care
WHO: World Health Organization
